# Letter from Dr. E. G. Betty

**Published:** 1882-07

**Authors:** E. G. Betty

**Affiliations:** Cincinnati


CINCINNATI, June 12th, 1882.

EDITOR REGISTER:—I know not whether the following be at all remarkable or not. It is, at all events, rather unusual, and with your permission I would like to put it on record: Master Frankie ——— is a nice, healthy little boy of six years of age. His teeth, above and below, are all well formed and strong, and consist only of the temporaries. The curious feature is, that he has *four* strong and perfectly developed superior *lateral* incisors, all standing in proper line, without any deviation. An accident somewhat nicked the right pair, but with that exception they are perfectly shaped. Order of development; Right and left inferior central incisors; same sup, centrals; right and left sup, lateral incisors; and then the supernumeraries. Respectfully,

E. G. Betty, D.D.S.

